# Poly[bis­(μ_3_-5-nitro­isophthalato)bis­(1,10-phenanthroline)dimanganese(II)]

**DOI:** 10.1107/S1600536809009052

**Published:** 2009-03-19

**Authors:** Hai-Dong Wang, Min-Min Li, Hong-Yin He, Fu-Bin Jiang

**Affiliations:** a Biological and Chemical Engineering School, Jiaxing University, Jiaxing 314001, People’s Republic of China; b College of Chemistry, Beijing Normal University, Beijing, People’s Republic of China

## Abstract

The title complex, [Mn_2_(C_8_H_3_NO_6_)_2_(C_12_H_8_N_2_)_2_]_*n*_, was synthesized under hydro­thermal conditions. The structure contains two independent Mn^II^ atoms, each coordinated in a distorted octa­hedral MnN_2_O_4_ geometry. [Mn_2_(phen)_2_] units (phen = 1,10-phenantroline) are bridged by 5-nitro­isophthalate (nip) ligands into ladder-like chains parallel to [100]. Adjacent polymeric chains are linked by C—H⋯O and π–π inter­actions [centroid-to-centroid distance = 3.6369 (12) Å] into a two-dimensional framework parallel to (010).

## Related literature

For related isophthalate complexes, see: He *et al.* (2004 ▶, 2005 ▶); Sun *et al.* (2003 ▶); Wu *et al.* (2002 ▶).
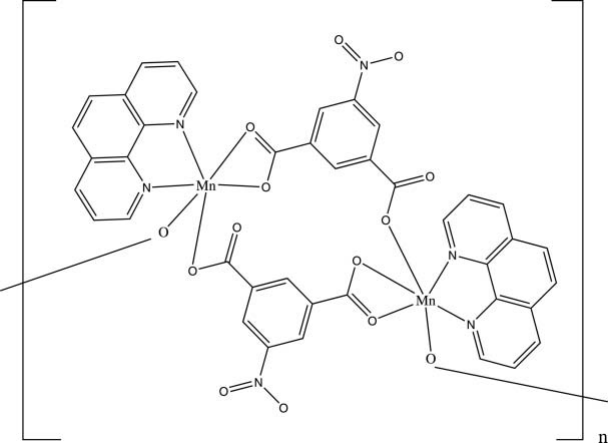


## Experimental

### 

#### Crystal data

[Mn_2_(C_8_H_3_NO_6_)_2_(C_12_H_8_N_2_)_2_]
*M* *_r_* = 888.52
Triclinic, 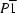

*a* = 10.0602 (1) Å
*b* = 14.3435 (2) Å
*c* = 14.6637 (2) Å
α = 104.052 (1)°
β = 102.633 (1)°
γ = 110.460 (1)°
*V* = 1812.69 (4) Å^3^
*Z* = 2
Mo *K*α radiation
μ = 0.78 mm^−1^
*T* = 293 K
0.28 × 0.23 × 0.19 mm

#### Data collection

Bruker SMART CCD diffractometer
Absorption correction: multi-scan (*SADABS*; Bruker, 2002 ▶) *T*_min_ = 0.82, *T*_max_ = 0.90
19255 measured reflections
6733 independent reflections
5771 reflections with *I* > 2σ(*I*)
*R*_int_ = 0.021

#### Refinement

*R*[*F* ^2^ > 2σ(*F* ^2^)] = 0.032
*wR*(*F* ^2^) = 0.082
*S* = 1.05
6733 reflections
541 parameters
H-atom parameters constrained
Δρ_max_ = 0.38 e Å^−3^
Δρ_min_ = −0.28 e Å^−3^

### 

Data collection: *SMART* (Bruker, 2002 ▶); cell refinement: *SAINT* (Bruker, 2002 ▶); data reduction: *SAINT*; program(s) used to solve structure: *SHELXS97* (Sheldrick, 2008 ▶); program(s) used to refine structure: *SHELXL97* (Sheldrick, 2008 ▶); molecular graphics: *ORTEP-3 for Windows* (Farrugia, 1997 ▶); software used to prepare material for publication: *PLATON* (Spek, 2009 ▶).

## Supplementary Material

Crystal structure: contains datablocks I, global. DOI: 10.1107/S1600536809009052/wm2223sup1.cif

Structure factors: contains datablocks I. DOI: 10.1107/S1600536809009052/wm2223Isup2.hkl

Additional supplementary materials:  crystallographic information; 3D view; checkCIF report

## Figures and Tables

**Table 1:** Selected bond lengths (Å)

Mn1—O6^i^	2.1498 (15)
Mn1—O11	2.1508 (15)
Mn1—N2	2.2531 (18)
Mn1—N1	2.2584 (18)
Mn1—O1	2.2661 (15)
Mn1—O2	2.2879 (15)
Mn2—O5	2.1608 (15)
Mn2—O12^ii^	2.1652 (15)
Mn2—O7	2.1938 (16)
Mn2—N5	2.2411 (18)
Mn2—N6	2.2697 (19)
Mn2—O8	2.3830 (16)

Symmetry codes: (i) 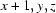; (ii) 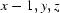.

**Table 2:** Hydrogen-bond geometry (Å, °)

*D*—H⋯*A*	*D*—H	H⋯*A*	*D*⋯*A*	*D*—H⋯*A*
C3—H3⋯O1^iii^	0.93	2.50	3.267 (4)	140
C10—H10⋯O2^iv^	0.93	2.54	3.210 (3)	129
C29—H29⋯O5	0.93	2.51	3.079 (3)	119
C31—H31⋯O3^v^	0.93	2.38	2.888 (4)	114
C38—H38⋯O7^vi^	0.93	2.45	3.121 (3)	129
C38—H38⋯O12^vii^	0.93	2.60	3.351 (3)	139

Symmetry codes: (iii) 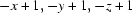; (iv) 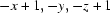; (v) 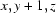; (vi) 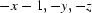; (vii) 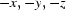.

**Fig. 1.**
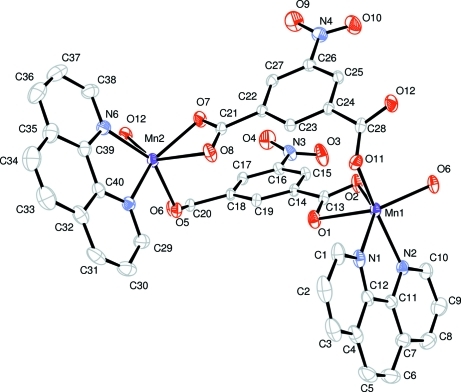
View of the title compound with the atom numbering scheme. Displacement ellipsoids for non-H atoms are drawn at the 30% probability level.

**Fig. 2.**
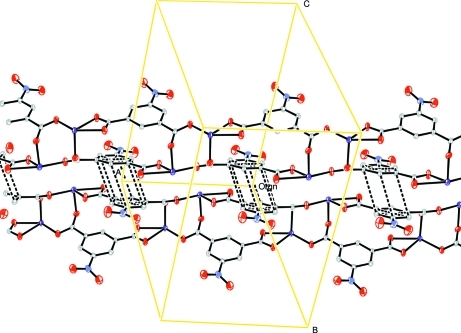
View of the π–π interactions between the nip ligands.

## supplementary crystallographic information

### Comment

Coordination polymers with isophthalate and its derivatives, such as 5-nitroisophthalate (He *et al.*, 2004), 5-sulfoisophthalate (Sun *et al.*, 2003), 5-hydroisophthalate (He *et al.*, 2005) or 5-aminoisophthalate (Wu *et al.*, 2002), have been attracted interest in recent years because of their potential applications and intriguing architectures with new topologies. Here we reported a novel compound, (I), [Mn_2_(nip)_2_(phen)_2_]_n_ (I), where nip = 5-nitroisophthalate and phen = 1,10-phenanthroline.

In the title compound (I), the coordination sphere of the two manganese ions can be best described as distorted octahedral (Fig.1). The carboxyl groups of the nip ligands with µ_2_-chelating coordination mode to the metal centres show an average Mn—O distance of 2.288 Å, whereas the carboxyl groups with a µ_1_-bridging mode have an average Mn—O distance of 2.158 Å. The Mn···Mn separation is 7.8156 (5) Å. A one-dimensional ladder-like framework is created by the bridging coordination mode of the nip ligands. Significant π–π stacking interactions exist between adjacent ladders, with a Cg10···Cg10(-x, -y, 1 - z) distance of 3.6369 (12) Å (Cg10 is the centroid of atoms C14–C19) (Fig. 2).

### Experimental

A mixture of MnSO_4_.H_2_O (0.0840 g, 0.5 mmol), 1,10-phenanthroline (0.1980 g, 1 mmol), 5-nitroisophthalic acid (0.2100 g, 1 mmol), 8 ml H_2_O and 8 ml EtOH was heated at 473 K for 5 d in a 20 ml Teflon-lined stainless-steel autoclave. After cooling, yellow plane-like crystals of the title compound were obtained.

### Refinement

The aromatic H atoms were generated geometrically, and were included in the refinements in the riding model approximation (C—H = 0.93 Å, *U*_iso_ = 1.2Ueq(C)).

### Crystal data

**Table:**

[Mn_2_(C_8_H_3_NO_6_)_2_(C_12_H_8_N_2_)_2_]	*V* = 1812.69 (4) Å^3^
*M**_r_* = 888.52	*Z* = 2
Triclinic, *P*1	*F*(000) = 900
Hall symbol: -P 1	*D*_x_ = 1.628 Mg m^−^^3^
*a* = 10.0602 (1) Å	Mo *K*α radiation, λ = 0.71073 Å
*b* = 14.3435 (2) Å	θ = 2.3–25.5°
*c* = 14.6637 (2) Å	µ = 0.78 mm^−^^1^
α = 104.052 (1)°	*T* = 293 K
β = 102.633 (1)°	Plane, yellow
γ = 110.460 (1)°	0.28 × 0.23 × 0.19 mm

### Data collection

**Table:**

Bruker SMART CCD diffractometer	6733 independent reflections
Radiation source: fine-focus sealed tube	5771 reflections with *I* > 2σ(*I*)
graphite	*R*_int_ = 0.021
φ and ω scans	θ_max_ = 25.5°, θ_min_ = 2.3°
Absorption correction: multi-scan (*SADABS*; Bruker, 2002)	*h* = −11→12
*T*_min_ = 0.82, *T*_max_ = 0.90	*k* = −17→16
19255 measured reflections	*l* = −17→17

### Refinement

**Table:**

Refinement on *F*^2^	Primary atom site location: structure-invariant direct methods
Least-squares matrix: full	Secondary atom site location: difference Fourier map
*R*[*F*^2^ > 2σ(*F*^2^)] = 0.032	Hydrogen site location: inferred from neighbouring sites
*wR*(*F*^2^) = 0.082	H-atom parameters constrained
*S* = 1.05	*w* = 1/[σ^2^(*F*_o_^2^) + (0.0319*P*)^2^ + 1.1304*P*] where *P* = (*F*_o_^2^ + 2*F*_c_^2^)/3
6733 reflections	(Δ/σ)_max_ < 0.001
541 parameters	Δρ_max_ = 0.38 e Å^−^^3^
0 restraints	Δρ_min_ = −0.28 e Å^−^^3^

### Special details

**Table:**

Geometry. All e.s.d.'s (except the e.s.d. in the dihedral angle between two l.s. planes) are estimated using the full covariance matrix. The cell e.s.d.'s are taken into account individually in the estimation of e.s.d.'s in distances, angles and torsion angles; correlations between e.s.d.'s in cell parameters are only used when they are defined by crystal symmetry. An approximate (isotropic) treatment of cell e.s.d.'s is used for estimating e.s.d.'s involving l.s. planes.
Refinement. Refinement of *F*^2^ against ALL reflections. The weighted *R*-factor *wR* and goodness of fit *S* are based on *F*^2^, conventional *R*-factors *R* are based on *F*, with *F* set to zero for negative *F*^2^. The threshold expression of *F*^2^ > σ(*F*^2^) is used only for calculating *R*-factors(gt) *etc*. and is not relevant to the choice of reflections for refinement. *R*-factors based on *F*^2^ are statistically about twice as large as those based on *F*, and *R*- factors based on ALL data will be even larger.

### Fractional atomic coordinates and isotropic or equivalent isotropic displacement parameters (Å^2^)

**Table:**

	*x*	*y*	*z*	*U*_iso_*/*U*_eq_	
O6	−0.38999 (16)	0.07873 (13)	0.39068 (12)	0.0419 (4)	
O12	0.48532 (16)	0.06651 (13)	0.14855 (12)	0.0448 (4)	
O10	0.0320 (2)	−0.28100 (15)	0.00966 (16)	0.0681 (6)	
O9	−0.1856 (2)	−0.28002 (15)	−0.02566 (18)	0.0761 (7)	
C40	−0.2137 (2)	0.40567 (17)	0.18610 (17)	0.0376 (5)	
C39	−0.3176 (2)	0.33731 (19)	0.08772 (17)	0.0389 (5)	
C38	−0.4691 (3)	0.1676 (2)	−0.02115 (18)	0.0501 (6)	
H38	−0.5058	0.0946	−0.0358	0.060*	
C37	−0.5213 (3)	0.2053 (3)	−0.0939 (2)	0.0665 (9)	
H37	−0.5929	0.1580	−0.1552	0.080*	
C36	−0.4671 (3)	0.3113 (3)	−0.0749 (2)	0.0695 (9)	
H36	−0.4999	0.3370	−0.1236	0.083*	
Mn1	0.50277 (3)	0.18469 (2)	0.40921 (2)	0.02943 (9)	
Mn2	−0.28274 (3)	0.18118 (3)	0.19550 (2)	0.03242 (9)	
O5	−0.22209 (16)	0.19531 (12)	0.35061 (11)	0.0364 (3)	
O11	0.46448 (15)	0.19055 (12)	0.26097 (11)	0.0372 (4)	
O1	0.28394 (16)	0.18994 (12)	0.41974 (13)	0.0444 (4)	
O8	−0.04288 (18)	0.19540 (13)	0.18704 (14)	0.0502 (4)	
N5	−0.18114 (19)	0.35909 (14)	0.25249 (13)	0.0350 (4)	
C14	0.0582 (2)	0.03847 (16)	0.38410 (14)	0.0278 (4)	
N1	0.60980 (19)	0.36391 (14)	0.47322 (15)	0.0375 (4)	
C17	−0.2442 (2)	−0.05564 (16)	0.36682 (15)	0.0314 (5)	
H17	−0.3447	−0.0876	0.3619	0.038*	
O2	0.29375 (16)	0.03586 (12)	0.38289 (13)	0.0439 (4)	
C22	−0.0057 (2)	0.03872 (16)	0.13375 (14)	0.0286 (4)	
C24	0.2403 (2)	0.04240 (16)	0.14906 (14)	0.0286 (4)	
N2	0.61892 (19)	0.23126 (13)	0.57383 (13)	0.0336 (4)	
C19	−0.0278 (2)	0.09562 (16)	0.38222 (15)	0.0281 (4)	
H19	0.0160	0.1661	0.3867	0.034*	
C21	−0.1018 (2)	0.09678 (18)	0.15297 (15)	0.0330 (5)	
C15	−0.0072 (2)	−0.06723 (16)	0.37599 (15)	0.0313 (4)	
H15	0.0485	−0.1071	0.3763	0.038*	
C18	−0.1786 (2)	0.04910 (16)	0.37374 (14)	0.0283 (4)	
C27	−0.0717 (2)	−0.06970 (17)	0.08307 (15)	0.0321 (5)	
H27	−0.1756	−0.1082	0.0612	0.038*	
C26	0.0202 (2)	−0.11973 (16)	0.06560 (15)	0.0315 (4)	
C25	0.1751 (2)	−0.06559 (17)	0.09654 (15)	0.0315 (5)	
H25	0.2340	−0.1010	0.0824	0.038*	
O7	−0.23937 (16)	0.04323 (13)	0.13500 (13)	0.0472 (4)	
C16	−0.1575 (2)	−0.11164 (16)	0.36741 (15)	0.0311 (4)	
C13	0.2228 (2)	0.09112 (17)	0.39602 (15)	0.0311 (5)	
C23	0.1499 (2)	0.09427 (16)	0.16730 (14)	0.0291 (4)	
H23	0.1942	0.1670	0.2024	0.035*	
C32	−0.1522 (3)	0.51645 (19)	0.2104 (2)	0.0501 (6)	
C11	0.6698 (2)	0.33550 (17)	0.62839 (17)	0.0367 (5)	
C28	0.4087 (2)	0.10391 (17)	0.18864 (16)	0.0330 (5)	
C20	−0.2706 (2)	0.11226 (17)	0.37167 (15)	0.0313 (5)	
N4	−0.0494 (2)	−0.23528 (16)	0.01253 (15)	0.0468 (5)	
N3	−0.2287 (2)	−0.22294 (15)	0.35983 (16)	0.0441 (5)	
C12	0.6636 (2)	0.40590 (17)	0.57451 (18)	0.0378 (5)	
N6	−0.3696 (2)	0.23105 (15)	0.06785 (14)	0.0385 (4)	
C10	0.6353 (3)	0.1663 (2)	0.62129 (19)	0.0439 (6)	
H10	0.6012	0.0950	0.5840	0.053*	
O4	−0.3650 (2)	−0.27042 (15)	0.32496 (17)	0.0670 (6)	
O3	−0.1460 (2)	−0.26194 (15)	0.39173 (19)	0.0714 (6)	
C1	0.6126 (3)	0.4296 (2)	0.4231 (2)	0.0499 (6)	
H1	0.5766	0.4019	0.3538	0.060*	
C4	0.7204 (3)	0.51567 (18)	0.6283 (2)	0.0519 (7)	
C2	0.6685 (3)	0.5404 (2)	0.4716 (3)	0.0638 (9)	
H2	0.6693	0.5846	0.4345	0.077*	
C30	−0.0212 (3)	0.5308 (2)	0.3741 (2)	0.0546 (7)	
H30	0.0433	0.5714	0.4392	0.066*	
C29	−0.0876 (3)	0.4210 (2)	0.34357 (18)	0.0446 (6)	
H29	−0.0652	0.3893	0.3894	0.054*	
C35	−0.3611 (3)	0.3823 (2)	0.0185 (2)	0.0549 (7)	
C7	0.7351 (3)	0.3753 (2)	0.73312 (19)	0.0490 (6)	
C8	0.7495 (3)	0.3033 (3)	0.7796 (2)	0.0589 (7)	
H8	0.7925	0.3266	0.8486	0.071*	
C3	0.7210 (3)	0.5818 (2)	0.5727 (3)	0.0640 (9)	
H3	0.7576	0.6546	0.6050	0.077*	
C31	−0.0517 (3)	0.5781 (2)	0.3078 (2)	0.0567 (8)	
H31	−0.0058	0.6517	0.3268	0.068*	
C9	0.7013 (3)	0.1998 (2)	0.7248 (2)	0.0579 (7)	
H9	0.7119	0.1519	0.7553	0.070*	
C5	0.7804 (3)	0.5524 (2)	0.7353 (2)	0.0667 (9)	
H5	0.8152	0.6242	0.7712	0.080*	
C34	−0.2953 (4)	0.4958 (3)	0.0461 (3)	0.0738 (9)	
H34	−0.3231	0.5260	0.0001	0.089*	
C6	0.7876 (3)	0.4866 (2)	0.7845 (2)	0.0639 (8)	
H6	0.8277	0.5136	0.8538	0.077*	
C33	−0.1950 (4)	0.5589 (2)	0.1364 (3)	0.0714 (9)	
H33	−0.1522	0.6320	0.1513	0.086*	

### Atomic displacement parameters (Å^2^)

**Table:**

	*U*^11^	*U*^22^	*U*^33^	*U*^12^	*U*^13^	*U*^23^
O6	0.0319 (8)	0.0502 (10)	0.0609 (10)	0.0265 (7)	0.0254 (7)	0.0257 (8)
O12	0.0266 (8)	0.0531 (10)	0.0569 (10)	0.0196 (7)	0.0187 (7)	0.0144 (8)
O10	0.0729 (13)	0.0406 (10)	0.0867 (15)	0.0330 (10)	0.0180 (11)	0.0098 (10)
O9	0.0470 (12)	0.0384 (11)	0.1048 (17)	0.0026 (9)	0.0096 (11)	−0.0006 (11)
C40	0.0331 (11)	0.0355 (12)	0.0498 (13)	0.0176 (10)	0.0188 (10)	0.0152 (10)
C39	0.0351 (12)	0.0439 (13)	0.0448 (13)	0.0198 (10)	0.0175 (10)	0.0190 (10)
C38	0.0410 (13)	0.0538 (15)	0.0413 (13)	0.0129 (12)	0.0096 (11)	0.0077 (11)
C37	0.0500 (16)	0.093 (2)	0.0393 (15)	0.0191 (16)	0.0061 (12)	0.0194 (15)
C36	0.0623 (19)	0.101 (3)	0.0581 (18)	0.0363 (19)	0.0194 (15)	0.0475 (18)
Mn1	0.02107 (15)	0.02731 (17)	0.04103 (19)	0.01103 (13)	0.01000 (13)	0.01304 (13)
Mn2	0.02599 (17)	0.03180 (18)	0.04039 (19)	0.01458 (14)	0.01036 (13)	0.01137 (14)
O5	0.0333 (8)	0.0370 (8)	0.0446 (9)	0.0201 (7)	0.0102 (7)	0.0181 (7)
O11	0.0267 (7)	0.0370 (8)	0.0413 (8)	0.0108 (7)	0.0039 (6)	0.0134 (7)
O1	0.0266 (8)	0.0334 (9)	0.0736 (12)	0.0121 (7)	0.0180 (8)	0.0190 (8)
O8	0.0402 (9)	0.0402 (10)	0.0769 (12)	0.0239 (8)	0.0249 (9)	0.0170 (9)
N5	0.0273 (9)	0.0353 (10)	0.0409 (10)	0.0133 (8)	0.0123 (8)	0.0099 (8)
C14	0.0232 (10)	0.0341 (11)	0.0315 (10)	0.0142 (9)	0.0111 (8)	0.0154 (9)
N1	0.0282 (9)	0.0327 (10)	0.0608 (12)	0.0155 (8)	0.0189 (9)	0.0242 (9)
C17	0.0226 (10)	0.0362 (12)	0.0368 (11)	0.0125 (9)	0.0116 (8)	0.0136 (9)
O2	0.0280 (8)	0.0415 (9)	0.0746 (12)	0.0215 (7)	0.0233 (8)	0.0252 (8)
C22	0.0246 (10)	0.0351 (11)	0.0287 (10)	0.0149 (9)	0.0091 (8)	0.0119 (9)
C24	0.0244 (10)	0.0361 (11)	0.0291 (10)	0.0150 (9)	0.0096 (8)	0.0139 (9)
N2	0.0292 (9)	0.0283 (9)	0.0435 (10)	0.0126 (8)	0.0107 (8)	0.0133 (8)
C19	0.0243 (10)	0.0307 (11)	0.0334 (10)	0.0135 (8)	0.0100 (8)	0.0149 (9)
C21	0.0272 (11)	0.0426 (13)	0.0310 (11)	0.0191 (10)	0.0082 (8)	0.0109 (9)
C15	0.0304 (10)	0.0336 (11)	0.0391 (11)	0.0194 (9)	0.0154 (9)	0.0159 (9)
C18	0.0248 (10)	0.0363 (11)	0.0291 (10)	0.0170 (9)	0.0101 (8)	0.0134 (9)
C27	0.0224 (10)	0.0378 (12)	0.0329 (11)	0.0099 (9)	0.0080 (8)	0.0127 (9)
C26	0.0327 (11)	0.0284 (11)	0.0318 (11)	0.0120 (9)	0.0102 (9)	0.0099 (9)
C25	0.0321 (11)	0.0399 (12)	0.0325 (11)	0.0225 (10)	0.0142 (9)	0.0154 (9)
O7	0.0229 (8)	0.0509 (10)	0.0599 (10)	0.0172 (7)	0.0119 (7)	0.0056 (8)
C16	0.0311 (11)	0.0279 (11)	0.0371 (11)	0.0129 (9)	0.0145 (9)	0.0129 (9)
C13	0.0235 (10)	0.0391 (12)	0.0369 (11)	0.0154 (9)	0.0110 (8)	0.0194 (9)
C23	0.0253 (10)	0.0319 (11)	0.0297 (10)	0.0126 (9)	0.0094 (8)	0.0094 (8)
C32	0.0480 (14)	0.0356 (13)	0.0738 (18)	0.0196 (11)	0.0302 (13)	0.0194 (13)
C11	0.0246 (10)	0.0307 (11)	0.0489 (13)	0.0094 (9)	0.0115 (9)	0.0082 (10)
C28	0.0242 (10)	0.0412 (12)	0.0401 (12)	0.0163 (9)	0.0111 (9)	0.0208 (10)
C20	0.0260 (10)	0.0379 (12)	0.0333 (11)	0.0188 (9)	0.0081 (8)	0.0117 (9)
N4	0.0497 (13)	0.0338 (11)	0.0506 (12)	0.0151 (10)	0.0138 (10)	0.0109 (9)
N3	0.0424 (12)	0.0325 (10)	0.0636 (13)	0.0160 (9)	0.0270 (10)	0.0187 (9)
C12	0.0248 (10)	0.0259 (11)	0.0588 (15)	0.0092 (9)	0.0148 (10)	0.0097 (10)
N6	0.0327 (10)	0.0395 (11)	0.0397 (10)	0.0141 (8)	0.0106 (8)	0.0111 (8)
C10	0.0426 (13)	0.0416 (13)	0.0533 (14)	0.0207 (11)	0.0156 (11)	0.0225 (11)
O4	0.0408 (11)	0.0435 (11)	0.1003 (16)	0.0024 (9)	0.0177 (10)	0.0266 (10)
O3	0.0638 (12)	0.0459 (11)	0.135 (2)	0.0354 (10)	0.0515 (13)	0.0480 (12)
C1	0.0404 (13)	0.0486 (15)	0.0824 (19)	0.0252 (12)	0.0301 (13)	0.0409 (14)
C4	0.0338 (12)	0.0274 (12)	0.089 (2)	0.0111 (10)	0.0233 (13)	0.0107 (13)
C2	0.0478 (15)	0.0481 (16)	0.128 (3)	0.0280 (13)	0.0463 (18)	0.0592 (19)
C30	0.0397 (14)	0.0488 (15)	0.0542 (16)	0.0086 (12)	0.0180 (12)	−0.0037 (13)
C29	0.0340 (12)	0.0486 (14)	0.0428 (13)	0.0132 (11)	0.0131 (10)	0.0083 (11)
C35	0.0543 (16)	0.0678 (18)	0.0600 (17)	0.0316 (14)	0.0250 (13)	0.0371 (14)
C7	0.0329 (12)	0.0509 (15)	0.0488 (14)	0.0114 (11)	0.0128 (11)	0.0039 (12)
C8	0.0495 (15)	0.080 (2)	0.0447 (15)	0.0256 (15)	0.0141 (12)	0.0220 (15)
C3	0.0438 (15)	0.0281 (13)	0.125 (3)	0.0159 (12)	0.0350 (17)	0.0273 (16)
C31	0.0475 (15)	0.0306 (13)	0.081 (2)	0.0108 (11)	0.0329 (14)	0.0002 (13)
C9	0.0571 (16)	0.0697 (19)	0.0569 (16)	0.0292 (15)	0.0191 (13)	0.0351 (15)
C5	0.0463 (16)	0.0359 (15)	0.088 (2)	0.0083 (12)	0.0192 (15)	−0.0120 (15)
C34	0.084 (2)	0.072 (2)	0.093 (2)	0.0406 (19)	0.035 (2)	0.058 (2)
C6	0.0556 (17)	0.0533 (17)	0.0577 (17)	0.0140 (14)	0.0161 (14)	−0.0060 (14)
C33	0.081 (2)	0.0463 (17)	0.105 (3)	0.0306 (16)	0.044 (2)	0.0396 (18)

### Geometric parameters (Å, °)

**Table:**

O6—C20	1.249 (3)	C24—C23	1.391 (3)
O6—Mn1^i^	2.1498 (15)	C24—C28	1.504 (3)
O12—C28	1.250 (3)	N2—C10	1.323 (3)
O12—Mn2^ii^	2.1652 (15)	N2—C11	1.357 (3)
O10—N4	1.216 (3)	C19—C18	1.390 (3)
O9—N4	1.216 (3)	C19—H19	0.9300
C40—N5	1.353 (3)	C21—O7	1.254 (3)
C40—C32	1.406 (3)	C15—C16	1.383 (3)
C40—C39	1.440 (3)	C15—H15	0.9300
C39—N6	1.357 (3)	C18—C20	1.505 (3)
C39—C35	1.400 (3)	C27—C26	1.383 (3)
C38—N6	1.322 (3)	C27—H27	0.9300
C38—C37	1.393 (4)	C26—C25	1.382 (3)
C38—H38	0.9300	C26—N4	1.472 (3)
C37—C36	1.355 (4)	C25—H25	0.9300
C37—H37	0.9300	C16—N3	1.467 (3)
C36—C35	1.405 (4)	C23—H23	0.9300
C36—H36	0.9300	C32—C31	1.405 (4)
Mn1—O6^ii^	2.1498 (15)	C32—C33	1.426 (4)
Mn1—O11	2.1508 (15)	C11—C7	1.409 (3)
Mn1—N2	2.2531 (18)	C11—C12	1.434 (3)
Mn1—N1	2.2584 (18)	N3—O4	1.216 (3)
Mn1—O1	2.2661 (15)	N3—O3	1.227 (3)
Mn1—O2	2.2879 (15)	C12—C4	1.414 (3)
Mn1—C13	2.600 (2)	C10—C9	1.399 (4)
Mn2—O5	2.1608 (15)	C10—H10	0.9300
Mn2—O12^i^	2.1652 (15)	C1—C2	1.415 (4)
Mn2—O7	2.1938 (16)	C1—H1	0.9300
Mn2—N5	2.2411 (18)	C4—C3	1.392 (4)
Mn2—N6	2.2697 (19)	C4—C5	1.437 (4)
Mn2—O8	2.3830 (16)	C2—C3	1.356 (4)
Mn2—C21	2.615 (2)	C2—H2	0.9300
O5—C20	1.261 (3)	C30—C31	1.356 (4)
O11—C28	1.263 (3)	C30—C29	1.384 (4)
O1—C13	1.251 (3)	C30—H30	0.9300
O8—C21	1.242 (3)	C29—H29	0.9300
N5—C29	1.322 (3)	C35—C34	1.435 (4)
C14—C19	1.385 (3)	C7—C8	1.399 (4)
C14—C15	1.388 (3)	C7—C6	1.431 (4)
C14—C13	1.509 (3)	C8—C9	1.353 (4)
N1—C1	1.324 (3)	C8—H8	0.9300
N1—C12	1.359 (3)	C3—H3	0.9300
C17—C16	1.377 (3)	C31—H31	0.9300
C17—C18	1.381 (3)	C9—H9	0.9300
C17—H17	0.9300	C5—C6	1.331 (4)
O2—C13	1.247 (2)	C5—H5	0.9300
C22—C27	1.383 (3)	C34—C33	1.337 (5)
C22—C23	1.389 (3)	C34—H34	0.9300
C22—C21	1.508 (3)	C6—H6	0.9300
C24—C25	1.383 (3)	C33—H33	0.9300
			
C20—O6—Mn1^i^	115.68 (14)	C17—C18—C19	119.77 (18)
C28—O12—Mn2^ii^	112.02 (14)	C17—C18—C20	119.94 (18)
N5—C40—C32	122.6 (2)	C19—C18—C20	120.29 (18)
N5—C40—C39	117.5 (2)	C22—C27—C26	118.63 (18)
C32—C40—C39	119.9 (2)	C22—C27—H27	120.7
N6—C39—C35	123.5 (2)	C26—C27—H27	120.7
N6—C39—C40	117.1 (2)	C25—C26—C27	122.58 (19)
C35—C39—C40	119.4 (2)	C25—C26—N4	118.62 (19)
N6—C38—C37	122.9 (3)	C27—C26—N4	118.80 (19)
N6—C38—H38	118.6	C26—C25—C24	118.43 (19)
C37—C38—H38	118.6	C26—C25—H25	120.8
C36—C37—C38	119.6 (3)	C24—C25—H25	120.8
C36—C37—H37	120.2	C21—O7—Mn2	94.70 (13)
C38—C37—H37	120.2	C17—C16—C15	122.94 (19)
C37—C36—C35	119.9 (3)	C17—C16—N3	117.91 (18)
C37—C36—H36	120.0	C15—C16—N3	119.14 (19)
C35—C36—H36	120.0	O2—C13—O1	122.15 (18)
O6^ii^—Mn1—O11	96.15 (6)	O2—C13—C14	119.64 (19)
O6^ii^—Mn1—N2	85.35 (6)	O1—C13—C14	118.21 (18)
O11—Mn1—N2	158.99 (6)	O2—C13—Mn1	61.62 (11)
O6^ii^—Mn1—N1	128.76 (6)	O1—C13—Mn1	60.62 (10)
O11—Mn1—N1	89.95 (6)	C14—C13—Mn1	177.21 (14)
N2—Mn1—N1	73.18 (7)	C22—C23—C24	120.76 (19)
O6^ii^—Mn1—O1	141.93 (6)	C22—C23—H23	119.6
O11—Mn1—O1	94.75 (6)	C24—C23—H23	119.6
N2—Mn1—O1	96.89 (6)	C31—C32—C40	116.7 (3)
N1—Mn1—O1	87.53 (6)	C31—C32—C33	124.4 (3)
O6^ii^—Mn1—O2	84.74 (6)	C40—C32—C33	118.9 (3)
O11—Mn1—O2	100.62 (6)	N2—C11—C7	122.2 (2)
N2—Mn1—O2	100.39 (6)	N2—C11—C12	117.1 (2)
N1—Mn1—O2	143.83 (6)	C7—C11—C12	120.6 (2)
O1—Mn1—O2	57.39 (5)	O12—C28—O11	123.81 (19)
O6^ii^—Mn1—C13	113.37 (6)	O12—C28—C24	118.37 (19)
O11—Mn1—C13	97.86 (6)	O11—C28—C24	117.83 (18)
N2—Mn1—C13	100.76 (6)	O6—C20—O5	124.43 (19)
N1—Mn1—C13	116.04 (7)	O6—C20—C18	117.77 (19)
O1—Mn1—C13	28.76 (6)	O5—C20—C18	117.80 (18)
O2—Mn1—C13	28.66 (6)	O9—N4—O10	123.5 (2)
O5—Mn2—O12^i^	97.51 (6)	O9—N4—C26	118.1 (2)
O5—Mn2—O7	98.78 (6)	O10—N4—C26	118.4 (2)
O12^i^—Mn2—O7	85.02 (6)	O4—N3—O3	123.7 (2)
O5—Mn2—N5	85.80 (6)	O4—N3—C16	118.8 (2)
O12^i^—Mn2—N5	130.44 (6)	O3—N3—C16	117.42 (19)
O7—Mn2—N5	143.60 (6)	N1—C12—C4	123.1 (2)
O5—Mn2—N6	152.61 (6)	N1—C12—C11	117.88 (19)
O12^i^—Mn2—N6	83.95 (7)	C4—C12—C11	119.0 (2)
O7—Mn2—N6	108.58 (7)	C38—N6—C39	117.6 (2)
N5—Mn2—N6	73.03 (7)	C38—N6—Mn2	126.66 (17)
O5—Mn2—O8	93.04 (6)	C39—N6—Mn2	115.68 (14)
O12^i^—Mn2—O8	141.58 (6)	N2—C10—C9	123.2 (2)
O7—Mn2—O8	56.82 (6)	N2—C10—H10	118.4
N5—Mn2—O8	86.99 (6)	C9—C10—H10	118.4
N6—Mn2—O8	102.76 (7)	N1—C1—C2	122.2 (3)
O5—Mn2—C21	95.56 (6)	N1—C1—H1	118.9
O12^i^—Mn2—C21	113.56 (7)	C2—C1—H1	118.9
O7—Mn2—C21	28.56 (6)	C3—C4—C12	116.9 (3)
N5—Mn2—C21	115.28 (7)	C3—C4—C5	124.3 (3)
N6—Mn2—C21	108.99 (7)	C12—C4—C5	118.6 (3)
O8—Mn2—C21	28.30 (6)	C3—C2—C1	119.5 (3)
C20—O5—Mn2	118.39 (13)	C3—C2—H2	120.3
C28—O11—Mn1	118.01 (13)	C1—C2—H2	120.3
C13—O1—Mn1	90.62 (12)	C31—C30—C29	119.0 (2)
C21—O8—Mn2	86.27 (12)	C31—C30—H30	120.5
C29—N5—C40	118.1 (2)	C29—C30—H30	120.5
C29—N5—Mn2	125.24 (17)	N5—C29—C30	123.3 (3)
C40—N5—Mn2	116.54 (14)	N5—C29—H29	118.3
C19—C14—C15	119.72 (18)	C30—C29—H29	118.3
C19—C14—C13	120.20 (18)	C39—C35—C36	116.5 (3)
C15—C14—C13	120.08 (18)	C39—C35—C34	119.0 (3)
C1—N1—C12	118.1 (2)	C36—C35—C34	124.5 (3)
C1—N1—Mn1	126.84 (18)	C8—C7—C11	117.3 (2)
C12—N1—Mn1	114.78 (14)	C8—C7—C6	124.3 (3)
C16—C17—C18	118.46 (18)	C11—C7—C6	118.4 (3)
C16—C17—H17	120.8	C9—C8—C7	120.4 (3)
C18—C17—H17	120.8	C9—C8—H8	119.8
C13—O2—Mn1	89.73 (12)	C7—C8—H8	119.8
C27—C22—C23	119.70 (19)	C2—C3—C4	120.2 (3)
C27—C22—C21	120.40 (18)	C2—C3—H3	119.9
C23—C22—C21	119.90 (18)	C4—C3—H3	119.9
C25—C24—C23	119.88 (18)	C30—C31—C32	120.2 (2)
C25—C24—C28	120.45 (18)	C30—C31—H31	119.9
C23—C24—C28	119.65 (18)	C32—C31—H31	119.9
C10—N2—C11	118.2 (2)	C8—C9—C10	118.7 (3)
C10—N2—Mn1	126.20 (15)	C8—C9—H9	120.6
C11—N2—Mn1	115.51 (14)	C10—C9—H9	120.6
C14—C19—C18	120.95 (19)	C6—C5—C4	121.9 (2)
C14—C19—H19	119.5	C6—C5—H5	119.0
C18—C19—H19	119.5	C4—C5—H5	119.0
O8—C21—O7	122.0 (2)	C33—C34—C35	121.4 (3)
O8—C21—C22	119.48 (18)	C33—C34—H34	119.3
O7—C21—C22	118.48 (19)	C35—C34—H34	119.3
O8—C21—Mn2	65.43 (11)	C5—C6—C7	121.4 (3)
O7—C21—Mn2	56.74 (11)	C5—C6—H6	119.3
C22—C21—Mn2	173.29 (16)	C7—C6—H6	119.3
C16—C15—C14	118.16 (19)	C34—C33—C32	121.4 (3)
C16—C15—H15	120.9	C34—C33—H33	119.3
C14—C15—H15	120.9	C32—C33—H33	119.3
			
N5—C40—C39—N6	−2.9 (3)	Mn1—O1—C13—C14	−177.10 (16)
C32—C40—C39—N6	178.1 (2)	C19—C14—C13—O2	−169.39 (19)
N5—C40—C39—C35	176.4 (2)	C15—C14—C13—O2	11.3 (3)
C32—C40—C39—C35	−2.6 (3)	C19—C14—C13—O1	11.1 (3)
N6—C38—C37—C36	1.3 (4)	C15—C14—C13—O1	−168.2 (2)
C38—C37—C36—C35	−1.2 (5)	C19—C14—C13—Mn1	−53 (3)
O12^i^—Mn2—O5—C20	40.49 (15)	C15—C14—C13—Mn1	127 (3)
O7—Mn2—O5—C20	−45.60 (15)	O6^ii^—Mn1—C13—O2	−2.45 (15)
N5—Mn2—O5—C20	170.76 (15)	O11—Mn1—C13—O2	97.78 (13)
N6—Mn2—O5—C20	131.83 (16)	N2—Mn1—C13—O2	−92.00 (13)
O8—Mn2—O5—C20	−102.49 (14)	N1—Mn1—C13—O2	−168.40 (13)
C21—Mn2—O5—C20	−74.21 (15)	O1—Mn1—C13—O2	−176.7 (2)
O6^ii^—Mn1—O11—C28	47.12 (15)	O6^ii^—Mn1—C13—O1	174.24 (12)
N2—Mn1—O11—C28	140.16 (18)	O11—Mn1—C13—O1	−85.53 (13)
N1—Mn1—O11—C28	176.14 (15)	N2—Mn1—C13—O1	84.69 (13)
O1—Mn1—O11—C28	−96.34 (15)	N1—Mn1—C13—O1	8.28 (15)
O2—Mn1—O11—C28	−38.66 (15)	O2—Mn1—C13—O1	176.7 (2)
C13—Mn1—O11—C28	−67.57 (15)	O6^ii^—Mn1—C13—C14	−120 (3)
O6^ii^—Mn1—O1—C13	−8.59 (18)	O11—Mn1—C13—C14	−20 (3)
O11—Mn1—O1—C13	97.70 (13)	N2—Mn1—C13—C14	151 (3)
N2—Mn1—O1—C13	−99.83 (13)	N1—Mn1—C13—C14	74 (3)
N1—Mn1—O1—C13	−172.56 (14)	O1—Mn1—C13—C14	66 (3)
O2—Mn1—O1—C13	−1.89 (12)	O2—Mn1—C13—C14	−117 (3)
O5—Mn2—O8—C21	96.07 (13)	C27—C22—C23—C24	1.1 (3)
O12^i^—Mn2—O8—C21	−10.06 (19)	C21—C22—C23—C24	−179.07 (18)
O7—Mn2—O8—C21	−2.43 (13)	C25—C24—C23—C22	0.3 (3)
N5—Mn2—O8—C21	−178.30 (14)	C28—C24—C23—C22	−178.33 (18)
N6—Mn2—O8—C21	−106.46 (14)	N5—C40—C32—C31	1.0 (3)
C32—C40—N5—C29	−0.1 (3)	C39—C40—C32—C31	180.0 (2)
C39—C40—N5—C29	−179.0 (2)	N5—C40—C32—C33	−178.8 (2)
C32—C40—N5—Mn2	−177.03 (17)	C39—C40—C32—C33	0.2 (4)
C39—C40—N5—Mn2	4.0 (2)	C10—N2—C11—C7	−1.7 (3)
O5—Mn2—N5—C29	18.08 (18)	Mn1—N2—C11—C7	175.40 (17)
O12^i^—Mn2—N5—C29	114.38 (18)	C10—N2—C11—C12	174.33 (19)
O7—Mn2—N5—C29	−81.0 (2)	Mn1—N2—C11—C12	−8.6 (2)
N6—Mn2—N5—C29	−179.51 (19)	Mn2^ii^—O12—C28—O11	−12.1 (3)
O8—Mn2—N5—C29	−75.20 (18)	Mn2^ii^—O12—C28—C24	168.14 (14)
C21—Mn2—N5—C29	−76.09 (19)	Mn1—O11—C28—O12	−95.4 (2)
O5—Mn2—N5—C40	−165.19 (15)	Mn1—O11—C28—C24	84.41 (19)
O12^i^—Mn2—N5—C40	−68.89 (17)	C25—C24—C28—O12	22.4 (3)
O7—Mn2—N5—C40	95.70 (17)	C23—C24—C28—O12	−159.0 (2)
N6—Mn2—N5—C40	−2.78 (15)	C25—C24—C28—O11	−157.41 (19)
O8—Mn2—N5—C40	101.53 (15)	C23—C24—C28—O11	21.2 (3)
C21—Mn2—N5—C40	100.64 (15)	Mn1^i^—O6—C20—O5	−9.5 (3)
O6^ii^—Mn1—N1—C1	106.59 (18)	Mn1^i^—O6—C20—C18	170.71 (13)
O11—Mn1—N1—C1	8.73 (18)	Mn2—O5—C20—O6	−92.3 (2)
N2—Mn1—N1—C1	176.01 (19)	Mn2—O5—C20—C18	87.53 (18)
O1—Mn1—N1—C1	−86.03 (18)	C17—C18—C20—O6	19.0 (3)
O2—Mn1—N1—C1	−99.4 (2)	C19—C18—C20—O6	−161.27 (19)
C13—Mn1—N1—C1	−90.01 (19)	C17—C18—C20—O5	−160.86 (19)
O6^ii^—Mn1—N1—C12	−79.94 (16)	C19—C18—C20—O5	18.9 (3)
O11—Mn1—N1—C12	−177.81 (14)	C25—C26—N4—O9	−170.6 (2)
N2—Mn1—N1—C12	−10.52 (14)	C27—C26—N4—O9	9.8 (3)
O1—Mn1—N1—C12	87.43 (15)	C25—C26—N4—O10	9.8 (3)
O2—Mn1—N1—C12	74.05 (18)	C27—C26—N4—O10	−169.8 (2)
C13—Mn1—N1—C12	83.46 (15)	C17—C16—N3—O4	21.2 (3)
O6^ii^—Mn1—O2—C13	177.74 (14)	C15—C16—N3—O4	−159.6 (2)
O11—Mn1—O2—C13	−86.96 (13)	C17—C16—N3—O3	−157.0 (2)
N2—Mn1—O2—C13	93.47 (13)	C15—C16—N3—O3	22.2 (3)
N1—Mn1—O2—C13	17.82 (19)	C1—N1—C12—C4	0.7 (3)
O1—Mn1—O2—C13	1.89 (12)	Mn1—N1—C12—C4	−173.37 (17)
O6^ii^—Mn1—N2—C10	−40.21 (18)	C1—N1—C12—C11	−175.89 (19)
O11—Mn1—N2—C10	−135.3 (2)	Mn1—N1—C12—C11	10.0 (2)
N1—Mn1—N2—C10	−173.1 (2)	N2—C11—C12—N1	−1.0 (3)
O1—Mn1—N2—C10	101.58 (18)	C7—C11—C12—N1	175.04 (19)
O2—Mn1—N2—C10	43.56 (19)	N2—C11—C12—C4	−177.75 (19)
C13—Mn1—N2—C10	72.73 (19)	C7—C11—C12—C4	−1.7 (3)
O6^ii^—Mn1—N2—C11	143.00 (15)	C37—C38—N6—C39	0.1 (4)
O11—Mn1—N2—C11	48.0 (3)	C37—C38—N6—Mn2	177.1 (2)
N1—Mn1—N2—C11	10.10 (14)	C35—C39—N6—C38	−1.5 (3)
O1—Mn1—N2—C11	−75.21 (15)	C40—C39—N6—C38	177.7 (2)
O2—Mn1—N2—C11	−133.23 (14)	C35—C39—N6—Mn2	−178.89 (19)
C13—Mn1—N2—C11	−104.06 (15)	C40—C39—N6—Mn2	0.4 (2)
C15—C14—C19—C18	0.9 (3)	O5—Mn2—N6—C38	−134.94 (19)
C13—C14—C19—C18	−178.42 (18)	O12^i^—Mn2—N6—C38	−40.3 (2)
Mn2—O8—C21—O7	4.2 (2)	O7—Mn2—N6—C38	42.4 (2)
Mn2—O8—C21—C22	−174.86 (17)	N5—Mn2—N6—C38	−175.9 (2)
C27—C22—C21—O8	−170.9 (2)	O8—Mn2—N6—C38	101.3 (2)
C23—C22—C21—O8	9.3 (3)	C21—Mn2—N6—C38	72.6 (2)
C27—C22—C21—O7	10.0 (3)	O5—Mn2—N6—C39	42.1 (2)
C23—C22—C21—O7	−169.81 (19)	O12^i^—Mn2—N6—C39	136.80 (16)
C27—C22—C21—Mn2	53.3 (13)	O7—Mn2—N6—C39	−140.54 (15)
C23—C22—C21—Mn2	−126.5 (12)	N5—Mn2—N6—C39	1.21 (15)
O5—Mn2—C21—O8	−86.09 (14)	O8—Mn2—N6—C39	−81.60 (16)
O12^i^—Mn2—C21—O8	173.20 (13)	C21—Mn2—N6—C39	−110.34 (16)
O7—Mn2—C21—O8	175.7 (2)	C11—N2—C10—C9	0.5 (3)
N5—Mn2—C21—O8	1.88 (15)	Mn1—N2—C10—C9	−176.24 (18)
N6—Mn2—C21—O8	81.57 (14)	C12—N1—C1—C2	−0.3 (3)
O5—Mn2—C21—O7	98.17 (14)	Mn1—N1—C1—C2	172.97 (17)
O12^i^—Mn2—C21—O7	−2.53 (15)	N1—C12—C4—C3	−0.6 (3)
N5—Mn2—C21—O7	−173.86 (13)	C11—C12—C4—C3	175.9 (2)
N6—Mn2—C21—O7	−94.16 (14)	N1—C12—C4—C5	−177.4 (2)
O8—Mn2—C21—O7	−175.7 (2)	C11—C12—C4—C5	−0.8 (3)
O5—Mn2—C21—C22	52.1 (12)	N1—C1—C2—C3	−0.2 (4)
O12^i^—Mn2—C21—C22	−48.6 (12)	C40—N5—C29—C30	−0.2 (3)
O7—Mn2—C21—C22	−46.1 (12)	Mn2—N5—C29—C30	176.50 (18)
N5—Mn2—C21—C22	140.0 (12)	C31—C30—C29—N5	−0.6 (4)
N6—Mn2—C21—C22	−140.3 (12)	N6—C39—C35—C36	1.6 (4)
O8—Mn2—C21—C22	138.2 (13)	C40—C39—C35—C36	−177.7 (2)
C19—C14—C15—C16	−0.8 (3)	N6—C39—C35—C34	−177.9 (2)
C13—C14—C15—C16	178.59 (19)	C40—C39—C35—C34	2.8 (4)
C16—C17—C18—C19	−0.7 (3)	C37—C36—C35—C39	−0.1 (4)
C16—C17—C18—C20	179.02 (18)	C37—C36—C35—C34	179.3 (3)
C14—C19—C18—C17	−0.2 (3)	N2—C11—C7—C8	1.6 (3)
C14—C19—C18—C20	−179.92 (18)	C12—C11—C7—C8	−174.3 (2)
C23—C22—C27—C26	−1.1 (3)	N2—C11—C7—C6	179.1 (2)
C21—C22—C27—C26	179.01 (19)	C12—C11—C7—C6	3.2 (3)
C22—C27—C26—C25	−0.2 (3)	C11—C7—C8—C9	−0.3 (4)
C22—C27—C26—N4	179.41 (19)	C6—C7—C8—C9	−177.6 (3)
C27—C26—C25—C24	1.6 (3)	C1—C2—C3—C4	0.2 (4)
N4—C26—C25—C24	−178.03 (19)	C12—C4—C3—C2	0.1 (4)
C23—C24—C25—C26	−1.6 (3)	C5—C4—C3—C2	176.7 (3)
C28—C24—C25—C26	177.04 (19)	C29—C30—C31—C32	1.7 (4)
O8—C21—O7—Mn2	−4.6 (2)	C40—C32—C31—C30	−1.9 (4)
C22—C21—O7—Mn2	174.50 (16)	C33—C32—C31—C30	177.9 (3)
O5—Mn2—O7—C21	−85.47 (14)	C7—C8—C9—C10	−0.8 (4)
O12^i^—Mn2—O7—C21	177.67 (14)	N2—C10—C9—C8	0.8 (4)
N5—Mn2—O7—C21	9.4 (2)	C3—C4—C5—C6	−174.7 (3)
N6—Mn2—O7—C21	95.77 (14)	C12—C4—C5—C6	1.9 (4)
O8—Mn2—O7—C21	2.41 (12)	C39—C35—C34—C33	−0.6 (5)
C18—C17—C16—C15	0.9 (3)	C36—C35—C34—C33	180.0 (3)
C18—C17—C16—N3	−179.89 (19)	C4—C5—C6—C7	−0.3 (4)
C14—C15—C16—C17	−0.2 (3)	C8—C7—C6—C5	175.1 (3)
C14—C15—C16—N3	−179.34 (19)	C11—C7—C6—C5	−2.3 (4)
Mn1—O2—C13—O1	−3.4 (2)	C35—C34—C33—C32	−1.9 (5)
Mn1—O2—C13—C14	177.14 (17)	C31—C32—C33—C34	−177.7 (3)
Mn1—O1—C13—O2	3.4 (2)	C40—C32—C33—C34	2.1 (4)

Symmetry codes: (i) *x*−1, *y*, *z*; (ii) *x*+1, *y*, *z*.

### Hydrogen-bond geometry (Å, °)

**Table:**

*D*—H···*A*	*D*—H	H···*A*	*D*···*A*	*D*—H···*A*
C3—H3···O1^iii^	0.93	2.50	3.267 (4)	140
C10—H10···O2^iv^	0.93	2.54	3.210 (3)	129
C29—H29···O5	0.93	2.51	3.079 (3)	119
C31—H31···O3^v^	0.93	2.38	2.888 (4)	114
C38—H38···O7^vi^	0.93	2.45	3.121 (3)	129
C38—H38···O12^vii^	0.93	2.60	3.351 (3)	139

Symmetry codes: (iii) −*x*+1, −*y*+1, −*z*+1; (iv) −*x*+1, −*y*, −*z*+1; (v) *x*, *y*+1, *z*; (vi) −*x*−1, −*y*, −*z*; (vii) −*x*, −*y*, −*z*.
